# A flow electrochemistry‐enabled synthesis of 2‐substituted *N*‐(methyl‐*d*)piperidines

**DOI:** 10.1002/jlcr.4006

**Published:** 2022-11-15

**Authors:** Azzam A. M. AL‐Hadedi, Stuart Sawyer, Stuart J. Elliott, Robert A. Green, Daniel J. O'Leary, Richard C. D. Brown, Lynda J. Brown

**Affiliations:** ^1^ Department of Chemistry, College of Science University of Mosul Mosul Iraq; ^2^ School of Chemistry University of Southampton Southampton UK; ^3^ Molecular Sciences Research Hub Imperial College London London UK; ^4^ Department of Chemistry Pomona College Claremont California USA

**Keywords:** flow electrosynthesis, long‐lived nuclear spin, monodeuterated, NMR

## Abstract

A synthesis of *N*‐monodeuteriomethyl‐2‐substituted piperidines is described. An efficient and readily scalable anodic methoxylation of *N*‐formylpiperidine in an undivided microfluidic electrolysis cell delivers methoxylated piperidine **3**, which is a precursor to a *N*‐formyliminium ion and enables C‐nucleophiles to be introduced at the 2‐position. The isotopically labelled *N*‐deuteriomethyl group is installed using the Eschweiler–Clarke reaction with formic acid‐*d*_2_ and unlabelled formaldehyde. Monodeuterated *N*‐methyl groups in these molecular systems possess small isotropic proton chemical shift differences important in the investigation of molecules that are able to support long‐lived nuclear spin states in solution nuclear magnetic resonance.

## Graphical abstract

Synthesis of *N*‐monodeuteriomethyl‐2‐substituted piperidines by efficient and scalable methoxylation of *N*‐formylpiperidine in an electrochemical flow cell, followed by an Eschweiler–Clarke reaction with formic acid‐*d*_2_ and unlabelled formaldehyde, is described. These monodeuterated *N*‐methyl possess small isotropic proton chemical shift differences important in the investigation of molecules that are able to support long‐lived nuclear spin states in solution NMR.

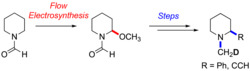

## 1 INTRODUCTION

Long‐lived nuclear spin order^1^, ^2^ of homonuclear spin‐1/2 pairs is protected from many of the relaxation mechanisms responsible for the short lifetimes of ordinary magnetisation, that is, the longitudinal relaxation time constant *T*_1_, and offers the possibility of designing molecules with unusually long relaxation times.^3^, ^4^, ^5^, ^6^, ^7^ The long‐lived state (LLS) of these molecules decays with the time constant *T*_s_, which can often be far longer than *T*_1_ because nuclear spin relaxation of long‐lived spin order is not dominated by the in‐pair dipole‐dipole relaxation mechanism in this case. This emerging technology has many exciting new applications^8^, ^9^, ^10^, ^11^, ^12^; however, to efficiently access LLSs with remarkably long lifetimes,^4^, ^10^, ^11^ the design and synthesis of suitable molecular systems is imperative.^7^

^13^C labelled methyl groups in specific molecules have been shown to support LLSs with lifetimes, which are significantly longer than *T*_1_.^13^, ^14^, ^15^ Despite the small, symmetrical nature of methyl groups, their exceptionally low rotational barrier in certain molecules^16^ allows for rapid rotation with respect to the rest of the molecule imposing approximate symmetry on the fluctuating nuclear spin interactions.^17^ Further to this, the monodeuteration of a methyl group within a chiral molecule can give rise to a small isotropic chemical shift difference between the geminal diastereotopic proton pair.^18^, ^19^, ^20^, ^21^, ^22^ An observable chemical shift difference, required to access the LLS in this case,^23^, ^24^, ^25^, ^26^ can only occur when there is (i) monodeuteration of the *N*‐methyl group; (ii) a local chiral environment for the CH_2_D protons; and (iii) suitably different populations for the three possible methyl rotamers.^18^, ^19^, ^20^, ^21^, ^22^, ^24^, ^25^, ^26^

It has been shown that the hyperconjugation effect of the nitrogen lone pair in *N*‐CH_2_D 2‐methylpiperdine is significant enough to cause an observable chemical shift difference between the two geminal protons.^18^, ^20^, ^21^, ^22^ Subsequent to this, a series of *N‐*CH_2_D 2‐substituted piperidines was investigated by computational and experimental methods to establish how the size and polarity of the substituent group could affect the 1,2‐stereoisomeric relationship and consequently, the strength of the rotational asymmetry within the CH_2_D group.^21^, ^22^ It was found that nonpolar and large 2‐substituent groups lead to appreciable chemical shift differences.^21^, ^22^

A convenient synthetic approach to generate *N*‐methyl piperidines that possess both a substituent in the 2‐position and a single deuterium label in the *N*‐methyl group is of interest to ongoing LLS investigations. Herein, we report a method for the synthesis of 2‐substituted *N‐*CH_2_D piperidines. The first step utilises flow electrosynthesis to provide the required methoxylated *N*‐formyl piperidine **3** in high yields and on demand, at scales commensurate with the desired application. The 2‐methoxylated piperidine **3** serves as a convenient *N*‐formyliminium ion precursor, which can react to allow different carbon nucleophiles to be introduced at the 2‐position as required. The final step exploits a modified Eschweiler–Clark reaction with formic acid‐*d*_*2*_ to selectively introduce the required singly deuterated methyl group.

## 2 RESULTS AND DISCUSSION

**FIGURE 1:**
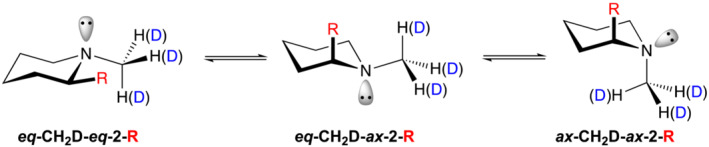
Stereoisomerism in *N‐*CH_2_D 2‐substituted piperidines **1** (R = Ph) and **2** (R = CCH)

**FIGURE 2:**
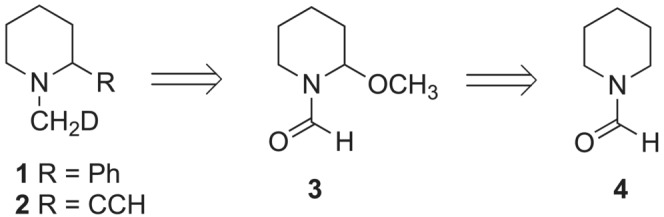
Approach to *N*‐CH_2_D 2‐substituted piperidine targets

From the group of 2‐substituted *N*‐CH_2_D piperidines that were identified as promising candidates to study LLSs, two compounds **1** and **2** required synthesis from a simple piperidine starting material. Calculations reported previously, revealed that the 2‐alkynyl and 2‐phenyl derivatives, **1** and **2**, favoured conformations with the substituent at the 2‐position adopting an axial orientation (Figure 1).^21^ The calculated mole fractions of the ***eq*‐CH**_**2**_ **D‐*ax*‐2‐R** conformers are 0.76 and 0.67 for **1** and **2**, respectively. In order to access piperidines **1** and **2** synthetically, 2‐methoxypiperidine‐1‐carbaldehyde (**3**) was considered to be a suitable intermediate, which could be prepared conveniently by flow electrosynthesis from piperidine‐1‐carbaldehyde (**4**) (Figure 2).

A versatile strategy to 2‐substituted piperidines involves oxidative α‐functionalisation of simple piperidines using chemical or electrochemical methods,^27^, ^28^, ^29^, ^30^ followed by reaction with C‐nucleophiles.^31^, ^32^, ^33^ The electrochemical, ‘reagent‐free’ approach is attractive in terms of reducing waste and avoiding hazards associated with stoichiometric chemical oxidants.^34^ Electrochemical flow cells with extended path lengths and small electrode gaps have been shown to be effective for anodic oxidation, achieving high conversions and productivities suitable for synthesis on a multi‐gram scale.^28^ In the present work, electrolysis in these extended path cells offers benefits such as high rates of production and selectivity, reduced requirement for supporting electrolyte compared to batch cells and flexibility to suit research laboratory applications at different scales.^30^, ^35^, ^36^, ^37^

**SCHEME 1:**
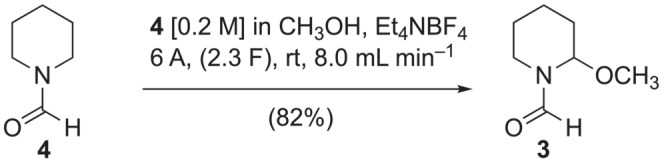
Anodic methoxylation of *N*‐formylpiperidine (**4**) on 0.2 M scale in flow

Our synthetic approach began with the anodic methoxylation of *N*‐formylpiperidine (**4**) on a 200 mmol scale (Scheme 1), which was carried out using an Ammonite 15 extended path flow electrolysis cell.^27^ The cell, which has been described previously, comprises of a carbon‐filled polymer (C‐PVDF) anode and steel cathode, with a spiral channel (length 200, width 0.5 cm and interelectrode gap of 0.75 mm) formed by a perfluoroelastomer (FFKM) gasket in a sandwich arrangement between the two electrodes. This gives a total electrode area of 100 cm^2^ and a reactor volume of 3.75 ml. The cell was fed with 1000 ml of a 0.2 M solution of *N*‐formylpiperidine (**4**) in MeOH containing 0.05 M Et_4_NBF_4_ as supporting electrolyte at a flow rate of 8.0 ml min^–1^ and cell current of 6.0 A (2.3 F). The methoxylated product **3** was formed in 82% isolated yield after purification by column chromatography, giving 23 g of the key intermediate **3** in just over 2‐h electrolysis time. The supporting electrolyte was easily recovered from the crude reaction solution simply by evaporation of solvent and precipitation from EtOAc, with recrystallisation giving pure Et_4_NBF_4_ suitable for reuse.

**SCHEME 2:**
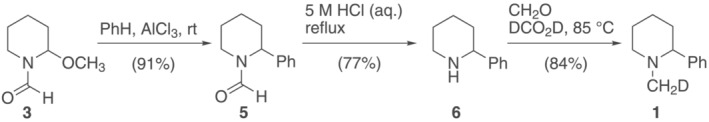
Synthesis of 1‐(methyl‐*d*)‐2‐phenylpiperidine (**1**)

With ample amounts of methoxylated piperidine **3** in hand, subsequent treatment of a benzene solution with AlCl_3_ afforded 2‐phenylpiperidine‐1‐carbaldehyde (**5**) in 91% yield (Scheme 2). Deprotection of the formyl group was achieved with aqueous HCl to provide piperidine **6** in good yield (77%).^27^, ^31^, ^38^, ^39^ The final step required introduction of the *N*‐CH_2_D group. Selective introduction of a monodeuterated methyl group presents a specific challenge. Full or partial deuteration of *N*‐methyl groups has been accomplished by a range of methods,^40^ including the reduction of *N*‐formyl piperidines using LiAlD_4_ to access CHD_2_ piperidines.^41^ In our synthesis, the classical Eschweiler–Clarke method^42^, ^43^, ^44^, ^45^ for the methylation of secondary amines was adapted by using formic acid‐*d*_*2*_ (98 atom %D) and unlabelled formaldehyde,^44^, ^45^ providing a convenient and reliable method to introduce the methyl group possessing a single deuterium in 84% yield with high specificity and deuterium incorporation (>95 atom %D, estimated by MS) (**1**, Scheme 2).

**SCHEME 3:**
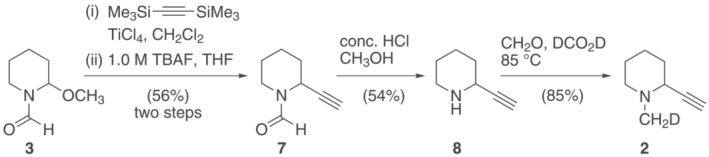
Synthesis of 2‐ethynyl‐1‐(methyl‐*d*)piperidine (**2**)

Methoxylated intermediate **3** also provides a convenient access to other substituted piperidines to investigate factors that influence the small chemical shift differences observed between diastereotopic protons in *N*‐CH_2_D groups.^21^, ^22^ This is exemplified by the synthesis of 2‐ethynyl‐1‐(methyl‐*d*)piperidine (**2**, Scheme 3), exploiting a Lewis acid promoted CC bond formation with bis (trimethylsilyl)acetylene followed by protodesilylation of the alkyne. As before, Eschweiler–Clarke reaction using formic acid‐*d*_*2*_ (98 atom %D) and unlabelled formaldehyde successfully introduced the required CH_2_D group (>95 atom %D, estimated by MS).

## 3 CONCLUSIONS

A synthesis of monodeuterated *N*‐methyl chiral piperidines is reported, which enables the study of small chemical shift differences between diastereotopic protons of their *N*‐CH_2_D groups and the derived long‐lived nuclear spin states. The synthesis relies on an efficient anodic α‐methoxylation of *N*‐formylpiperidine (**8**) and application of the methoxylated intermediate **3** in CC bond‐forming reactions with carbon nucleophiles and specific monodeuterio‐*N*‐methylation using an Eschweiler–Clarke reaction. Access to >23 g of the methoxylated intermediate **3** was conveniently achieved using flow electrolysis in an undivided electrochemical flow cell at ambient temperature, in a short time and without the need for chemical oxidising agents.

## 4 EXPERIMENTAL

### 4.1 General experimental

Chemicals including anhydrous solvents and isotopically enriched materials were purchased from Merck and used as supplied. All reactions were peformed under inert atmospheres in oven‐dried glassware. ^1^H and ^13^C NMR spectra were recorded in CDCl_3_ or D_2_O solution using a Bruker DPX 400 spectrometer (400 and 101 MHz, respectively). All NMR spectra were reprocessed using ACD/Labs software. Chemical shifts are reported using CHCl_3_ as an internal standard (δ 7.27 ppm ^1^H, δ 77.36 ppm ^13^C, respectively). Infrared spectra were recorded on a Nicolet 380 spectrometer fitted with a Diamond platform, as solids or neat liquids. Electron impact (EI) low‐resolution mass spectra were recorded on a Trace 2000 Series GC‐MS. Electrospray (ES) low‐resolution mass spectra were recorded on a Waters ZMD or Waters TQD quadrupole spectrometer.

The Ammonite 15 electrolysis cell is available from Cambridge Reactor Design.^46^ The cell has been described previously,^27^ comprising a carbon‐filled polymer (C‐PVDF) anode and steel cathode, with a spiral channel (length 200 cm, width 0.5 cm and interelectrode gap of 0.75 mm) formed by a perfluoroelastomer (FFKM) gasket in a sandwich arrangement between the two electrodes. The total working electrode area is 100 cm^2^, and reactor volume is 7.5 ml. Instructions for assembly and use of the Ammonite electrochemical flow cells can be found in the user guide.^46^ In the present work, both working and counter electrodes were cleaned and polished before use; organic contaminant was removed by washing with MeOH and then gentle polishing with cotton wool and silica (99.8%, pore size 60 Å, 230–400 mesh particle size, purchased from Merck). For the C/PVDF electrode care should be taken to maintain a flat surface. The C/PVDF electrode was polished using a polishing cloth stacked on a flat support, which was wetted with MeOH before adding silica on the surface of the polishing cloth. The surface of the electrode was polished using a circular motion, taking care to only remove the minimum amount of material to give a clean surface. Finally, the electrode was rinsed with MeOH to remove the residue of any carbon and silica, before allowing it to dry.

Once assembled, the Ammonite reactor is connected to the pump, and the reaction solvent is pumped through to ensure leak‐free operation and confirm the pump is providing the correct flow rate. The Ammonite cell is then connected to the power supply via crocodile clips, and the power supply is turned on at the required current, and pumping of the reaction solution through the cell is commenced. The effluent is collected in a suitable flask and worked‐up as described in the experimental procedure. On completion of the reaction, the reactor should be dismantled, and all the components cleaned to avoid parts becoming stuck together with consequent risk of breakages.

### 4.2 1‐(Methyl‐*d*)‐2‐phenylpiperidine (**1**)

To 2‐phenylpiperidine (1.00 g, 6.21 mmol) was added formaldehyde (1.51 ml of 37% in H_2_O, 18.6 mmol, 3.0 equiv.) followed by careful addition of formic acid‐*d*_2_ (1.17 ml of 95% in D_2_O, 98 atom %D, 31.0 mmol, 5.0 equiv.). The reaction was heated at 85°C (using a water bath) for 4 h before being cooled to rt. Water (2 ml) was added, and the acidic aqueous reaction was extracted with petroleum ether. The aqueous layer was basified to pH 12 using 6 M NaOH and extracted with Et_2_O (×5). The combined Et_2_O extractions were dried (Na_2_SO_4_) and concentrated on a rotary evaporator without vacuum (bath temp = 40**°**C). This gave the title compound as a yellow oil (921 mg, 5.23 mmol, 84%, >95 atom %D by HRMS). ^1^H NMR (400 MHz, CDCl_3_) δ 7.28–7.18 (m, 5H) 2.99 (br d, 1H, *J* = 11.6 Hz), 2.71 (dd, 1H, *J =* 11.0, 3.0 Hz), 2.06 (m, 1H), 1.93 (s, 2H), 1.83–1.12 (m, 6H) ppm; ^13^C NMR (101 MHz, CDCl_3_) δ 144.9, 128.4, 127.4, 127.0, 71.2, 57.5, 45.6 (t, *J*_D,C_ = 20.5 Hz, CH_2_D), 35.9, 26.2, 25.0 ppm; MS ESI^+^ (m/z) 177.3 [M + H]^+^; HRMS (ES^+^) for C_12_H_17_DN calculated 177.1497, found 177.1499 Da. The intensity ratio for the molecular ions [C_12_H_17_DN]^+^ (177.1497) and [C_12_H_18_N]^+^ (176.1430) were 99:1, showing >95% monodeuteration.

### 4.3 (*N*‐CH_2_D)‐2‐Ethynylpiperidine (**2**)

To 2‐ethynylpiperidine (70 mg, 0.64 mmol) was added formaldehyde (157 μl of 37 wt. % in H_2_O, 58 mg, 1.93 mmol, 3.0 equiv.) followed by careful addition of formic acid‐*d*_2_ (120 μl of 95% in D_2_O, 98 atom %D, 3.20 mmol, 5.0 equiv.), and the reaction was heated at 85°C (using a water bath) for 3 h. The reaction was cooled to rt, water (1 ml) added and the acidic aqueous reaction was extracted with petroleum ether. The aqueous layer was basified to pH 12 using 6 M NaOH and extracted with Et_2_O (×5). The combined Et_2_O extractions were dried (MgSO_4_) and concentrated on a rotary evaporator without vacuum (bath temp = 40**°**C) to give the title compound as a pale yellow oil (67 mg, 0.54 mmol, 85%, >95 atom %D by HRMS). ^1^H NMR (400 MHz, CDCl_3_) δ 3.38 (m, 1H), 2.56 (m, 1H), 2.37–2.27 (m, 4H), 1.87–1.71 (m, 2H), 1.68–1.42 (m, 4H) ppm; ^13^C NMR (101 MHz, CDCl_3_) δ 77.2, 73.5, 67.0, 53.8, 43.9 (t, *J*_D,C_ = 20.5 Hz, CH_2_D), 31.5, 25.6, 20.5 ppm; MS EI (m/z) 124.0 [M^+•^] (20%); HRMS (ES^+^) for C_8_H_13_DN calculated 125.1184, found 125.1183 Da.

### 4.4 2‐Methoxypiperidine‐1‐carbaldehyde (**3**)

Prior to assembly of the reactor, the working electrode (carbon‐filled polyvinylidene fluoride) was polished (see general experimental). A solution piperidine‐1‐carbaldehyde (**4**, 22.6 g, 0.200 mol, 0.2 M) in MeOH (1 L) and electrolyte Et_4_ NBF_4_ (10.8 g, 50.0 mmol, 0.05 M) was passed through an electrochemical cell (Ammonite 15, manufactured by Cambridge Reactor Design^27^, ^46^) at a flow rate of 8 ml min^–1^ and cell current of 6 A. The product was concentrated in vacuo. The resulting white solid was treated with EtOAc and filtered to recover the electrolyte (reused after recrystallization from MeOH and dried overnight under vacuum at 90°C). The filtrate was concentrated in vacuo to afford the crude product as colourless oil, purified by silica gel column chromatography, eluting with EtOAc (100%) provided the methoxylated product **3** as a colourless oil (23.5 g, 0.164 mol, 82%). ^1^H NMR data are consistent with those previously reported (at 60 MHz, CDCl_3_ ^47^). The product exhibited rotamers in its ^1^H NMR spectrum. ^1^H NMR (400 MHz, CDCl_3_) δ 8.10 (0.3H, s), 8.09 (0.7H, s), 5.46 (0.3H, m), 4.52 (0.7H, t, *J* = 3.0 Hz) 4.15 (0.7H, m), 3.26 (0.6H, m), 3.20 (1H, s) 3.14 (2H, s), 2.69 (0.7H, td, *J* = 13.0 Hz, 3.3 Hz), 1.98–1.30 (6H, m) ppm; ^13^C NMR (101 MHz, CDCl_3_) δ 162.1, 161.2, 85.6, 78.2, 55.0, 54.0, 41.7, 35.8, 31.1, 29.8, 25.9, 24.4, 19.2, 19.1 ppm; MS ESI^+^ (m/z) 166.1 [M + Na]^+^.

### 4.5 2‐Phenylpiperidine‐1‐carbaldehyde (**5**)

A solution of 2‐methoxypiperidine‐1‐carbaldehyde **3** (200 mg, 1.40 mmol) in anhydrous benzene (2 ml) was added to a suspension of AlCl_3_ (559 mg, 4.19 mmol) in anhydrous benzene (2 ml) at rt. The mixture was stirred for 20 min at rt. The reaction was diluted with brine (3 ml), and the organic phase was separated, re‐extracting the aqueous phase with EtOAc (4 × 5 ml). The combined organics were dried (MgSO_4_) and concentrated in vacuo to afford the title product **5** as a colourless oil (242 mg, 1.28 mmol, 91%). Characterisation data are consistent with those previously reported.^48^ The product exhibited rotamers in its ^1^H NMR spectrum. ^1^H NMR (400 MHz, CDCl_3_) δ 8.28 (0.5H, s), 8.03 (0.5H, s), 7.41–7.24 (5H, m) 5.76 (0.5H, d, *J* = 5.3 Hz), 4.76 (0.5H, t, *J* = 4.3 Hz), 4.10 (0.5H, m), 3.46 (0.5H, m) 3.09 (0.5H, td, *J* = 13.1, 3.5 Hz), 2.96 (0.5H, td, *J* = 13.1, 3.5 Hz), 2.43 (1H, m) 1.89 (1H, m), 1.76‐1.50 (4H, m) ppm; ^13^C NMR (101 MHz, CDCl_3_) δ 162.0, 161.7, 138.4, 138.0, 128.9, 128.6, 128.3, 126.7, 126.6, 126.5, 57.1, 49.5, 43.0, 37.9, 29.5, 26.9, 26.4, 25.0, 20.7, 19.9 ppm; MS ESI^+^ (m/z) 190.1 [M + H]^+^.

### 4.6 2‐Phenylpiperidine (**6**)

A solution of 2‐phenylpiperidine‐1‐carbaldehyde **5** (110 mg, 0.58 mmol) in 5 M HCl _aq_. (1 ml) was heated under reflux for 23 h. Following complete hydrolysis of the amide, 5 M NaOH _aq_. was added to raise the pH to 10–12 and a white solid formed. The solid was removed by filtration and washed with H_2_O (×3) and dried in vacuo (72.3 mg, 0.448 mmol, 77%). Characterisation data are consistent with those previously reported.^49^ ^1^H NMR (400 MHz, CDCl_3_) δ 7.38–7.17 (5H, m), 3.87 (1H, br s), 3.58 (1H, dd, *J* = 11.1, 2.5 Hz), 3.12 (1H, br d, *J* = 11.5), 2.73 (1H, td, *J* = 11.5, 3.9 Hz), 1.89–1.74 (2H, m), 1.52–1.40 (4H, m) ppm; ^13^C NMR (101 MHz, CDCl_3_) δ 143.6, 128.1, 127.0, 126.5, 61.8, 47.0, 33.7, 24.8, 24.7 ppm; MS ESI^+^ (m/z) 162.2 [M + H]^+^.

### 4.7 2‐Ethynylpiperidine‐1‐carbaldehyde (**7**)

A solution of TiCl_4_ (14.7 ml of 1.0 M in CH_2_Cl_2_, 14.7 mmol) was cooled to 0°C, and a solution of bis (trimethylsilyl)acetylene (1.53 g, 9.00 mmol) in dry CH_2_Cl_2_ (10 ml) was added dropwise, and then the resulting solution was stirred for a further 10 min at 0°C. 2‐Methoxypiperidine‐1‐carbaldehyde (1.00 g, 6.98 mmol) in dry CH_2_Cl_2_ (6 ml) was then added to the reaction dropwise at 0°C. The reaction was allowed to warm to rt, then stirred for 16 h and then poured slowly on a saturated solution of potassium sodium tartrate _(aq.)_ at 0°C. Sat. NaHCO_3 (aq.)_ was added until pH 10 and the aqueous solution extracted with Et_2_O (×3). Combined organic layers washed with brine and dried (MgSO_4_) and concentrated in vacuo. This crude yellow oil was dissolved in 1.0 M TBAF in THF (10 ml) and stirred at rt for 1.5 h. The reaction was diluted with water, the pH raised to 10 with sat. NaHCO_3 (aq.)_ and then the aqueous extracted with Et_2_O (×3). Combined organics were washed with brine, dried (MgSO_4_) and concentrated in vacuo. Purification by silica gel column chromatography (20%–40% EtOAc: hexane) gave the title compound as a clear oil (534 mg, 2.06 mmol, 56%). ^1^H NMR (400 MHz, CDCl_3_) δ 8.12 (0.4H, s), 7.95 (0.6H, s), 5.38 (0.6H, m), 4.46 (0.4H, m), 4.05 (0.4H, dt, *J* = 13.3, 3.7 Hz), 3.44–3.40 (1.2H, m), 3.09 (0.4H, ddd, *J* = 13.3, 11.9, 3.4 Hz), 2.43 (0.4H, d, *J* = 2.3 Hz), 2.27 (0.6H, d, *J* = 2.3 Hz), 1.94–1.58 (5H, m), 1.44–1.36 (1H, m) ppm; ^13^C NMR (101 MHz, CDCl_3_) δ 160.6, 160.4, 80.9, 80.4, 74.0, 72.1, 47.1, 42.8, 40.0, 37.3, 31.8, 29.9, 26.1, 24.6, 20.8, 20.3 ppm; MS EI (m/z) 137.1 [M^+•^], 108 [M‐CHO^+•^].

### 4.8 2‐Ethynylpiperidine (**8**)

To 2‐ethynylpiperidine‐1‐carbaldehyde **7** (200 mg, 1.46 mmol) in methanol (4 ml) was added conc. HCl (1 ml, 11.6 mmol) and stirred at room temperature for 18 h. The reaction was concentrated in vacuo, and the product recrystallised from EtOH. To obtain the free amine, the above salt was dissolved in water and 5 M NaOH added dropwise to raise the pH to 10 and then extracted with Et_2_O (×5). The combined organics were dried (MgSO_4_) and concentrated in vacuo (no heat) to give the desired amine **8** as a clear oil (86 mg, 0.79 mmol, 54%).^1^H NMR (400 MHz, CD_3_OD, data for the HCl salt) δ 4.22 (1H, m), 3.41–3.34 (2H, m), 3.12 (1H, m, *J* = 13.0, 9.5, 3.7 Hz) 2.17–2.09 (1H, m), 1.96–1.62 (5H, m); ^13^C NMR (101 MHz, CDCl_3_) δ 77.8, 76.9, 45.7, 41.6, 28.4, 21.8, 19.4, ppm.

## Data Availability

The data that support the findings of this study are available from the corresponding author upon reasonable request
